# Functional Characteristics and Coping Strategies among Rugby Athletes: A Cluster Analysis Approach

**DOI:** 10.3390/jpm14030292

**Published:** 2024-03-09

**Authors:** Walter Sapuppo, Davide Giacconi, Vincenzo Monda, Antonietta Messina, Salvatore Allocca, Sergio Chieffi, Mariateresa Ricci, Ines Villano, Daniele Saccenti, Claudia Maria Mineo, Margherita Boltri, Marcellino Monda, Girolamo Di Maio, Antonietta Monda, Marco La Marra

**Affiliations:** 1 Department of Psychology, Sigmund Freud University Wien, 20143 Milan, Italy; 2 Studi Cognitivi, Cognitive Psychotherapy School and Research Center, 20121 Milan, Italy; 3 Department of Economics, Law, Cybersecurity, and Sports Sciences, University of Naples “Parthenope”, 80133 Naples, Italy; 4 Department of Precision Medicine, University of Campania “Luigi Vanvitelli”, 80138 Naples, Italy; 5 Department of Experimental Medicine, University of Campania “Luigi Vanvitelli”, 80138 Naples, Italy; 6 Department of Wellness, Nutrition and Sport, Telematic University Pegaso, 80143 Naples, Italy; 7 Istituto di Ricovero e Cura a Carattere Scientifico (I.R.C.C.S.) Istituto Auxologico Italiano, Experimental Laboratory for Metabolic Neurosciences Research, 28824 Piancavallo, Italy; 8 Department of Psychology, Università Cattolica del Sacro Cuore, 20123 Milan, Italy; 9 Department for the Promotion of Human Science and Quality of Life, Telematic University San Raffaele, 00166 Rome, Italy

**Keywords:** cognitive flexibility, cognitive fusion, coping strategies, mental health, physical activity, rugby, sport, athletes

## Abstract

The developing domain of mental health in sports has gained much interest, acknowledging its pivotal role in athlete performance and well-being. The aim of this research is to provide a quantitative description concerning the levels of mental health, physical activity, cognitive fusion, cognitive flexibility, and coping strategies that characterize rugby athletes by using a data-driven approach. A total of 92 rugby athletes took part in this study and filled out a set of self-administered questionnaires. A correlational analysis showed that general well-being was positively associated with years spent playing rugby (*r* = 0.23) and coping mechanisms (*r* = 0.29). Athletes’ well-being was also negatively correlated with cognitive inflexibility (*r* = −0.41) and cognitive fusion (*r* = −0.39). A k-means cluster analysis identified two unique groups: group 1, characterized by higher levels of psychological well-being, lower levels of physical activity, greater cognitive flexibility, improved coping techniques, and reduced cognitive fusion, and group 2, which exhibits opposite characteristics. The discrepancies observed in psychological characteristics such as coping strategies, cognitive fusion, and cognitive inflexibility highlight their potential impact on the general health of rugby players. To comprehend the complex interplay between psychological and physical elements in rugby athletes, long-term studies with larger samples are crucial.

## 1. Introduction

The subject of mental health in sports is becoming more relevant and receiving greater focus from sports psychologists, athletes, coaches, and sports clubs. The World Health Organization (WHO) has defined mental health, referring to a condition of optimal psychological well-being, characterized by an individual’s awareness and utilization of their abilities, effective management of everyday pressures, productive work performance, and active participation in their community. The reason for such interest lies in the fact that empirical research has consistently shown that emotional and cognitive factors play a crucial role in influencing athletes’ performance and career trajectory [1,2,3,4,5]. Indeed, through the integration of physical, cognitive, and emotional aspects, athletes can attain the intended degree of psychological equilibrium and optimize their performance [6,7,8].

Physical activity encompasses any slight bodily movement that results in energy expenditure for the individual. The significance of physical activity is substantial as it has the potential to offset athletes’ adverse encounters and enhance their bodily and mental well-being [5,9,10,11,12,13]. Engaging in regular physical activity yields numerous bodily benefits, such as the prevention of various health-threatening conditions (e.g., cardiovascular issues, high blood pressure, type 2 diabetes) [14,15,16,17,18]. Additionally, it significantly influences mood and mental well-being [19,20,21,22,23]. Furthermore, particularly in adolescents, engaging in physical exercise is crucial as it facilitates the enhancement of motor abilities, such as coordination, agility, explosiveness, dynamic and static balance, and the growth of muscular mass [24,25,26,27,28,29,30].

Turning to athletes’ cognitive processes, cognitive fusion and cognitive flexibility emerge as crucial elements in terms of their influence on athletes’ mental states and performance outcomes [31]. Cognitive flexibility is widely recognized as an important cognitive ability that can significantly contribute to successful adaptation to changes in the environment [32,33,34]; cognitive fusion, conversely, denotes the state in which individuals erroneously “merge” with their ideas and emotions, impeding their ability to regulate their behaviors [35]. In the sport psychology research strand, cognitive fusion is often referred to as the occurrence of excessive self-criticism, self-doubt, or intrusive distractions that might hinder an athlete’s capacity to concentrate and perform optimally, ultimately resulting in burnout. Cognitive flexibility serves as an opposing force to the construct previously mentioned by prioritizing the improvement of cognitive skills [36,37,38,39]. This cognitive ability allows for nearly simultaneous multitasking, creative or divergent thinking, and the ability to shift perspectives [40]. Studies conducted on team athletes have highlighted that players who demonstrate higher levels of cognitive flexibility correlate with the experience of enhanced performance [40]. Cognitive flexibility, as defined here, pertains to a wide range of modifications in behavior rather than specific reactions. It enables individuals to effectively manage both external and internal stressors [41]. These cognitive abilities play a crucial role in both healthy and unhealthy behavior and are closely linked to the functions of the prefrontal lobes [42,43,44,45]. Strong interpersonal abilities positively influence the mental, emotional, and physical health of athletes in both direct and indirect ways. Hence, the significance of cognitive flexibility in achieving individual objectives is purportedly founded [41]. In sports that involve tactical reaction patterns, such as rugby, soccer, or basketball, cognitive flexibilities seem to play a pivotal role, since they enable individuals to assess and adjust to new situations with greater speed and effectiveness [41]. Test outcomes of accomplished athletes can be extrapolated to encompass general cognitive domains, enabling comparisons with the average performance of the general population. An effective team player can be identified by their exceptional spatial attention, split attention, working memory, and mentalizing skills. The individual must possess the ability to swiftly adjust, alter tactics, and suppress reactions. These qualities are commonly known as “game intelligence”, referred to synergistically as executive functions in neuropsychology [46,47,48,49,50,51,52,53,54,55,56]. Athletes encounter several obstacles throughout their careers, such as physical ailments, intense competition, declining performance, and the need to maintain a balance between personal and professional commitments. Coping methods are therefore crucial to the way in which athletes handle these difficulties and uphold their overall well-being and performance [57]. Coping strategies encompass the intellectual and behavioral actions that individuals employ to effectively handle or adjust to challenging circumstances, pressures, or requirements [2]. In the field of sport psychology, coping refers to the various psychological, emotional, and behavioral reactions that players employ to manage the pressures associated with their athletic pursuits [2,58]. Observing problem-focused coping methods is crucial due to their beneficial impact on athletes’ health and their effectiveness in addressing burnout and stress in general [59]. These coping techniques involve the athlete’s deliberate actions to directly manage or alter the stressor or scenario that is producing distress [60]. Athletes often employ problem-focused coping mechanisms such as goal planning, problem solving, seeking social support, time management, and seeking professional aid [61]. Emotion-focused coping techniques represent another category of coping strategies; these techniques encompass endeavors to control emotions and handle emotional suffering linked to the stressor [57,62]. Emotion-focused coping strategies employed by athletes include several tactics such as relaxation methods, constructive self-dialogue, mental imagery, mindfulness practices, and seeking social support to find solace in emotional distress [2,63]. 

To date, the scientific literature has deepened the role of the abovementioned cognitive and behavioral processes in heterogenous populations of athletes (e.g., soccer players, tennis players, basketball players, combat athletes, swimmers, and skiers) [64,65,66,67,68,69,70,71], but not specifically among rugby practitioners. Therefore, this research aims to provide a quantitative description concerning the levels of mental health, physical activity, cognitive fusion, cognitive flexibility, and coping strategies that characterize rugby athletes by using a data-driven approach.

## 2. Materials and Methods

### 2.1. Participants

**Figure 1:**
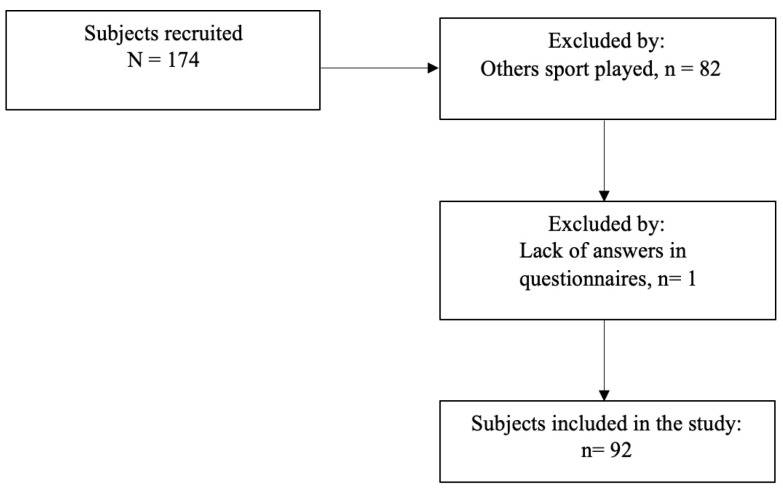
Flow chart: inclusions of study participants (February–April 2023).

Participants were recruited on a voluntary basis from the general population and through contact with rugby players and clubs. The subjects were recruited online through platforms such as social media and email, and completed the questionnaires online. The study focused on adult subjects aged 18 years or older who have participated in sports at a competitive or amateur level, either currently or in the past. From the initial sample of 174 individuals, 92 rugby athletes were identified and included in this study. It is worth noting that only one subject was excluded due to incomplete questionnaire responses. For further details on the inclusion criteria, please refer to Figure 1.

### 2.2. Study Design and Procedure

This is a non-clinical cross-sectional study conducted between February and April 2023. Individuals who met the inclusion criteria were kindly requested to provide their informed consent for their data to be processed for scientific and research purposes. The purpose of this study, as well as the strict confidentiality of data collection and analysis, were thoroughly explained to the participants. The document, questionnaires, and methods of data collection and storage were approved by the Ethics Committee of Sigmund Freud University, Ethics Commission of the Faculty of Psychotherapy Science and the Faculty of Psychology. The reference for this approval is GCP4Q7JFBO3P6I90070.

### 2.3. Data Collection

For the purpose of this cross-sectional study, a 24-item questionnaire was created to investigate (1) the level of physical activity, (2) the type of sport practiced, and (3) the frequency and duration of sports activity. Basic information such as nationality, age, gender, weight, and height was also collected to provide a more precise description of the surveyed population. Participants completed six self-administered questionnaires online, which took approximately 20 min. The questionnaires used were the Short Form of International Physical Activity Questionnaire Italian version (IPAQ-7) [72]; the Short-Form Health Survey Italian version (SF-36) [73]; The Acceptance and Action Questionnaire Italian version (AAQ-II) [74]; the Cognitive Fusion Questionnaire Italian version (I-CFQ) [75]; and the Coping Orientation to Problems Experienced Inventory Italian version (Brief-COPE) [76].

### 2.4. Psychometric Instruments

Short Form of International Physical Activity Questionnaire Italian version (IPAQ-7) [72]: This 7-item questionnaire aims to measure the type and amount of physical activity an individual usually performs. Questions refer to physical activity during the past 7 days at work, moving from place to place, and during leisure time.

Short-Form Health Survey Italian version (SF-36) [73]: This self-report questionnaire is designed to assess health-related quality of life and health status. The questionnaire consists of 36 questions that can be divided into 8 scales: physical functioning (10 items), psychological well-being (5 items), general health perception (5 items), limitations due to physical health (4 items), energy and dissatisfaction (4 items), limitations due to emotional issues (3 items), social engagement (2 items), and pain (2 items).

The Acceptance and Action Questionnaire Italian version (AAQ-II) [74]: This is a 10-item self-administered questionnaire and represents a basic instrument for assessing cognitive flexibility, and is used on an international scale for both clinical and research purposes. The items are evaluated on a scale ranging from 1 to 7, where 1 represents “never true” and 7 represents “always true”. Greater psychological rigidity, experience avoidance, and possible psychological distress are all indicated by higher overall AAQ-II scores. More psychological flexibility is indicated by lower overall scores.

Cognitive Fusion Questionnaire Italian version (I-CFQ) [75]: This is a 7-item self-report questionnaire intended to identify cognitive fusion, which is the process of considering one’s thoughts as literally and objectively true. Each item is rated on a 7-point Likert scale ranging from 1 (never) to 7 (always), and the total score is calculated by aggregating all factors. Higher total scores indicate greater cognitive fusion.

Coping Orientation to Problems Experienced Inventory Italian version (Brief-COPE) [76]: This is a 25-item self-report questionnaire divided into 5 scales, each consisting of 2 items. The questionnaire is scored from 1 to 4, ranging from “Not at all” to “Very much”, and is designed to measure effective and ineffective coping mechanisms for stressful life events. Every scale is examined independently: (1) avoidance strategies, (2) transcendent orientation, (3) positive attitude, (4) social support, (5) problem orientation.

### 2.5. Data Analysis

Descriptive statistics were calculated on the sample in the first instance. Subsequently, correlational analyses were performed on physical activity, cognitive fusion, coping strategies, cognitive inflexibility, and general health. Log transformation was used to achieve better normal distributions of data when values of skewness and kurtosis exceeded the conventional parameters of the Gaussian curve [77]. A *k*-means cluster analysis was then computed to find distinctive subject groups based on weekly physical activity, cognitive inflexibility, coping strategies, cognitive fusion, and general health. A canonical discriminant analysis was conducted to examine potential multivariate group differences. Additionally, univariate group differences were assessed for significance using unpaired two-sample *t*-tests or Wilcoxon rank sum tests, depending on the distribution of the variables. Normality was evaluated using the Shapiro–Wilk test and visual inspection of Q-Q plots. The last step of the analysis was to outline the descriptive statistics regarding the groups identified as a result of the clustering procedure. The statistical significance cut-off level was set at *p* < 0.05, 2-tailed. Data analysis was performed using statistical software, including SPSS for macOS (Version 28.0), R (Version 4.3.1), and RStudio for macOS (Version 2023.12.1).

## 3. Results

### 3.1. Sample Descriptive Statistics

The characteristics of the study participants (N = 92), with respect to their age, gender, nationality, body mass index (BMI), and physical activity-related aspects, are outlined in Table 1.

### 3.2. Correlations among Physical Activity, General Health, Coping Strategies, Cognitive Fusion, and Cognitive Flexibility

Several zero-order bivariate correlations were calculated to explore the potential relationship between physical activity, general health, coping strategies, cognitive fusion, and cognitive flexibility in the sample. Variables whose distribution did not approach statistical normality were log-transformed to be included in these analyses [77]. Statistically significant positive correlations were observed between participants’ levels of well-being and their coping capacities (*r*(90) = 0.29, *p* < 0.01). The number of years athletes spent practicing rugby positively correlated with their levels of well-being (*r*(90) = 0.23, *p* < 0.05). Negative moderate correlations were detected between general health and cognitive fusion (*r*(90) = −0.39, *p* < 0.001), as well as between general health and cognitive inflexibility (*r*(90) = −0.41, *p* < 0.001). A comprehensive portrait of the results of this analysis is shown in Table 2.

### 3.3. Sample Clustering

**Figure 2:**
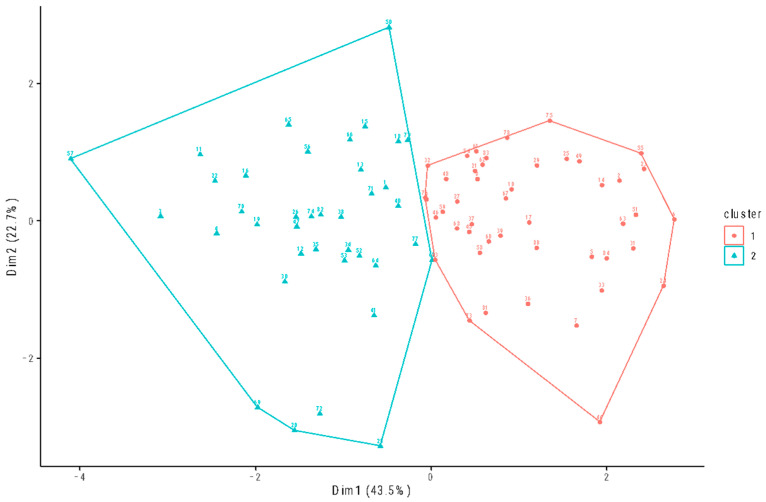
Cluster plot depicting the two groups of rugby players extracted by *k*-means cluster analysis (i.e., group 1, in red; group 2, in blue). The majority of the variance is likely explained by the first two principal components, which are utilized to plot the data points. The first dimension explains 43.5% of the variance, while the second dimension explains 22.7% of the variance. The two dimensions were identified following a principal component analysis automatically performed by the R function fviz_cluster.

A k-means cluster analysis was conducted to investigate the relationship between weekly physical activity, cognitive flexibility, coping strategies, cognitive fusion, and general health among rugby players. The analysis resulted in the identification of two distinct groups (group 1 and group 2), as shown in Figure 2.

Before computing the cluster, it was necessary to standardize each variable. Furthermore, it was necessary to exclude eight subjects from the analysis due to missing values. Once the *z* scores were extracted, an estimation of the optimal number of clusters (*k*) was performed by making use of two methods. According to the average silhouette method, the optimal number of clusters, *k*, is the value that maximizes the average silhouette over a range of possible values of *k* [78]. The gap statistic method has been employed to compare the overall intra-cluster variation for various values of k with their expected values under the null reference distribution of the data [79]. 

**Figure 3:**
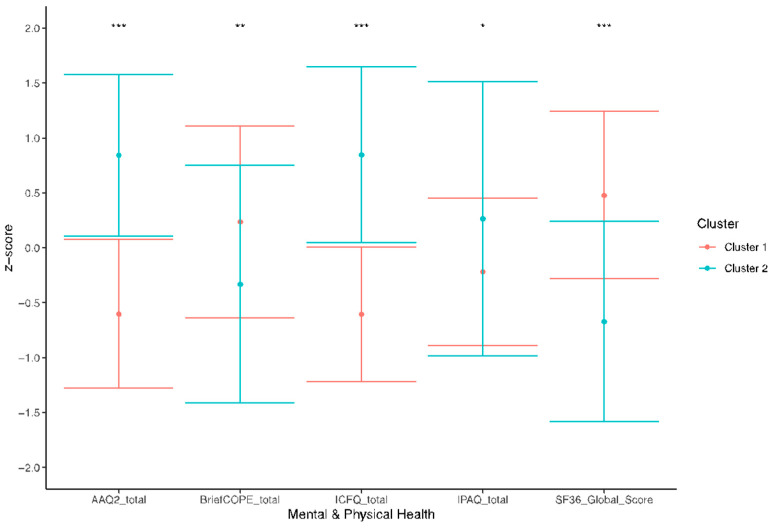
Scatter plot representing the means ± standard deviation of the standardized scores obtained by the two groups of rugby players yielded by the *k*-means cluster analysis. The variables considered are as follows: weekly physical activity (IPAQ_total), cognitive inflexibility (AAQ2_total), coping strategies (BriefCOPE_total), cognitive fusion (ICFQ_total), and general health (SF36_Global_Score). * = *p* < 0.05; ** = *p* < 0.01; *** = *p* < 0.001.

According to the results of the canonical discriminant analysis, there were notable distinctions between the two groups of rugby players that were identified through the clustering procedure (canonical discriminant function summary: canonical correlation: 0.83, Wilks’s Lambda = 0.31, *χ*^2^(5, N = 84) = 93, *p* < 0.001). Overall, 97.8% of the first group and 100% of the second group were classified correctly, yielding an overall correct classification of 98.8% by weekly physical activity, cognitive flexibility, coping strategies, cognitive fusion, and general health. The canonical discriminant function exhibited the strongest correlation with the discriminant variables in relation to cognitive fusion (*r*(82) = 0.76). Univariate differences between the two groups of rugby players were also assessed in the discriminant analysis. Unpaired two-sample *t*-tests highlighted a statistically significant difference in weekly physical activity (*t*(82) = 2.25, *p* < 0.05), cognitive flexibility (*t*(82) = 8.52, *p* < 0.001), coping strategies (*t*(82) = 2.80, *p* < 0.01), cognitive fusion (*t*(82) = 9.03, *p* < 0.001), and general health (*t*(82) = 6.71, *p* < 0.001) between the groups. See Figure 3 for a graphical representation.

### 3.4. Clusters Description

Overall, the first group of rugby players (i.e., group 1) showed high cognitive flexibility, high coping strategies, low cognitive fusion, low weekly physical activity, and high general health. In contrast, the second group (i.e., group 2) featured low cognitive flexibility, low coping strategies, high cognitive fusion, high weekly physical activity, and low general health (for a graphical representation, see Figure 2 and Figure 3). The Wilcoxon rank sum tests showed no significant differences between the two groups of rugby players regarding BMI and years of regular physical activity practice, but concerning age (*p* < 0.001) and years of rugby practice (*p* < 0.05), higher levels were observed in group 1. No statistically significant associations were found between group and gender. Socio-demographic data as well as unstandardized values of weekly physical activity, cognitive flexibility, coping strategies, cognitive fusion, and general health are reported in Table 3.

## 4. Discussion

The present study aims to provide a quantitative description concerning the levels of mental health, physical activity, cognitive fusion, cognitive flexibility, and coping strategies that characterize rugby athletes by using a data-driven approach. Given the lack of scientific studies regarding rugby athletes specifically, these findings provide insight into the complex interaction between these elements and define specific characteristics within the rugby player group.

Upon examining the descriptive statistics, 53.3% of participants reported that they participate in physical activity to enhance both their bodily and mental well-being. This result highlights the shift in individuals’ attention towards the significance of mental well-being rather than solely focusing on performance. Such awareness likely assumes significant importance in the field of sports psychology, as certain athletes endure repercussions on their mental and physical well-being from a life defined by the pursuit of sporting excellence [1]. If the drive to enhance athletic performance and engage in competition exhibits comparable patterns, it is likely to suggest that, for these athletes, (1) the need for challenge and the need for excellence are inherently interconnected and (2) competition may play a crucial role in motivating individuals to enhance their athletic ability [80,81,82].

Consistent with the existing literature [19,83], the correlational analyses conducted in this study highlight that those athletes who reported higher levels of general health also showed higher levels of physical activity, enhanced coping capacities, elevated cognitive flexibility, and reduced cognitive fusion. In addition, cognitive flexibility may impact adherence and performance in physical activity, hence increasing the motivation for people of all age groups to participate in physical endeavors. Cognitive flexibility may facilitate individuals in adjusting more readily to alterations in exercise regimens, approaching obstacles with a more receptive mindset, and rebounding more swiftly from setbacks or losses, thus promoting the sustenance of a physically active lifestyle over an extended period. The correlation between physical activity and cognitive flexibility has been documented throughout several age groups, spanning from adulthood to old age. Existing research has established a correlation between engaging in physical activity and various executive functions, including but not limited to selective attention, task switching, the inhibition of prepotent responses, and working memory capacity [84,85,86,87,88,89,90,91,92,93,94]. This indicates the positive impact of physical activity on cognitive flexibility throughout one’s entire lifespan [95,96,97]. Such a strong association between physical activity and cognitive flexibility highlights the need to view exercise not only as a means of preserving physical fitness but also as a valuable means of enhancing mental health and well-being [98,99,100,101,102,103,104].

The application of k-means clustering revealed the presence of two separate clusters within the population of rugby players, with each cluster exhibiting distinct psychological and physical characteristics. Group 1 displayed a profile that suggested higher psychological well-being and lower levels of physical activity in comparison to group 2. Our results also indicate that group 1 exhibited higher cognitive flexibility, increased use of coping techniques, decreased cognitive fusion, higher scores in general health, and lower levels of weekly physical activity. In contrast, group 2 exhibited lower cognitive flexibility, reduced utilization of coping techniques, increased cognitive fusion, worse overall health scores, and higher levels of weekly physical activity. The disparities revealed in psychological factors such as coping mechanisms, cognitive fusion, and cognitive flexibility highlight their potential influence on the general health of rugby players. The utilization of coping mechanisms and reduced cognitive fusion in group 1 may enhance their ability to handle stress, thereby leading to improved performance and health outcomes. On the other hand, the increased cognitive inflexibility and cognitive fusion in group 2 may make them more prone to responding poorly to stressors, which could have a negative impact on their performance and health. It is crucial to note that the participation of young athletes in a competitive sports environment does not result in the same experience for all individuals. Different young athletes may undergo unique emotional and cognitive changes throughout regular-season games [105]. The contrasting levels of physical activity among the two groups emphasize the complex correlation between exercise patterns and psychological well-being among rugby players. Group 1’s lower physical activity levels may appear contradictory to health outcomes, but they could be indicative of deliberate rest intervals or customized training programs [106,107]. On the other hand, the greater levels of physical activity in group 2 may imply a more intense training program, but they could also suggest excessive training or behavior to cope with psychological problems [108,109,110]. Hence, when examining young athletes engaged in competitive play, it is plausible that the intensity of their training impacts the emotions and thoughts they experience because of the daily biological, social, and psychological challenges they encounter, as well as the pressure to attain “victory at any expense” [105,111,112,113]. Analyzing the psychological characteristics of each group, this study suggests that rugby players in group 1, who exhibited elevated levels of cognitive flexibility, deflation, and coping, showed higher levels of mental well-being compared to those in group 2, who displayed lower coping abilities and cognitive flexibility, but high cognitive fusion. The characteristics displayed in group 1 could enable athletes to make rapid decisions and readily adapt to unexpected changes that occur during gameplay; individuals with enhanced cognitive flexibility also have the ability to effectively navigate field problems and promptly respond to unforeseen circumstances, resulting in decreased stress levels and enhanced mental well-being.

Regarding coping strategies, individuals in group 1, who demonstrated elevated levels of adaptability, might possess a broader repertoire of tactics to effectively address the problems and obstacles they face in rugby, both within and outside the playing area. These can encompass problem-solving techniques, interpersonal assistance, and emotional self-control, all of which are crucial for effectively handling the pressures of athletics and sustaining a sound mental equilibrium. In contrast, individuals belonging to group 2, who exhibited low coping abilities and high degrees of inflexibility and cognitive fusion, may experience an unfavorable state in terms of general well-being. Inadequate coping mechanisms for stress and inflexibility of the mind can impair their ability to adjust to the demands of rugby and handle the stressors linked to competition, hence heightening their vulnerability to mental health issues like anxiety and depression [114,115].

Overall, the results seem to validate the significance of cognitive flexibility and coping abilities in enhancing the mental health and overall well-being of rugby players. Athletes who exhibit these traits are likely to be more prone to confront the demands of their sport, sustaining a favorable psychological state. Conversely, individuals with limited proficiency in these abilities may be more susceptible to emotional health concerns. This emphasizes the relevance of promoting the development of these abilities through supportive and instructional efforts to enhance the psychological well-being of athletes [116,117,118,119,120].

Analyzing the psychological characteristics of rugby players might provide valuable insights for implementing specific interventions to enhance performance and overall mental health. Interventions that prioritize improving coping techniques and decreasing cognitive fusion may be advantageous for group 2 players to alleviate the negative impact of stress on their performance and well-being.

The present study suffers from the following main limitations: this study’s cross-sectional design restricts the ability to establish causality, highlighting the necessity for longitudinal investigations to elucidate temporal associations. Furthermore, the omission of some variables (i.e., Brief Cope’s “*Transcendental Orientation*” sub-scale, IPAQ total score, years spent practicing regular physical activity, and years spent practicing rugby) because of non-normal distributions may have disregarded possible correlations. To address these limitations and gain a more comprehensive picture of the psychological and physical profiles of rugby players, future research could utilize further data-driven statistical methods or increase the size and the heterogeneity of the sample. Implementing these changes may enhance the study’s contributions to the field by providing a more thorough understanding of the dynamics and potentially identifying causal relationships.

## 5. Conclusions

According to this study, in order to gain a comprehensive understanding of rugby players’ overall well-being, it is important to consider psychological factors in addition to physical attributes. This research identified eight necessary characteristics that are required to establish a conducive atmosphere for fostering positive development. The list includes various criteria to ensure physical and psychological well-being, establishing a clear and consistent framework with appropriate adult oversight, fostering supportive relationships, providing opportunities for individuals to feel a sense of belonging, promoting positive social norms, offering support for personal effectiveness and significance, facilitating skill development, and integrating the efforts of families, schools, and communities. This study’s findings could be useful in the development of interventions and supportive strategies aimed at improving the physical and mental outcomes of players. Furthermore, this research has identified distinct profiles among this group of athletes, which could inform more focused approaches for intervention and support.

## Figures and Tables

**Table 1:** Summary of the sample characteristics.

Variable	Mean ± Standard Deviation, or Frequency (%)
Age, years	33.01 ± 13.52
Gender	
Male	83 (90.2%)
Female	9 (9.8%)
Nationality	
Italian	90 (97.8%)
British	1 (1.1%)
French	1 (1.1%)
Prevailing motivation related to the practice of physical activity	
Competition	15 (16.3%)
Entertainment, social occasions	11 (12.0%)
Improvement in body image	2 (2.2%)
Improvement in physical and psychological health	49 (53.3%)
Improvement in athletic performance	15 (16.3%)
Body Mass Index	28.08 ± 4.17
Time spent practicing regular physical activity, years	18.74 ± 10.17
Time spent practicing rugby, years	14.28 ± 8.65

**Table 2:** Zero-order bivariate correlations.

	Years Practicing Regular Physical Activity	Years Practicing Rugby	IPAQ Total	ICFQ Total	BC Total	AAQ2 Total	SF36 Global Score
Years Practicing Regular Physical Activity	1.00						
Years Practicing Rugby	0.472 ***	1.00					
IPAQ Total	0.017	−0.082	1.00				
ICFQ Total	−0.051	−0.184	0.140	1.00			
BC Total	0.044	0.167	0.040	−0.127	1.00		
AAQ2 Total	−0.171	−0.198	0.129	0.783 ***	−0.155	1.00	
SF36 Global Score	0.073	0.229 *	0.058	−0.391 ***	0.289 **	−0.405 ***	1.00

Note. ICFQ = Italian Cognitive Fusion Questionnaire; BC = Coping Orientation to Problems Experienced Inventory Italian version; AAQ2 = Acceptance and Action Questionnaire Italian version; SF36 = Short-Form Health Survey Italian version; * = *p* < 0.05; ** = *p* < 0.01; *** = *p* < 0.001.

**Table 3:** Summary of the group characteristics after *k*-means clustering.

Variable	Group 1, Mean ± Standard Deviation, or Frequency (%)	Group 2, Mean ± Standard Deviation, or Frequency (%)
n	46 (54.76%)	38 (45.24%)
Age, years	37.61 ± 13.31	27.05 ± 11.10
Gender		
Males	44 (95.70%)	32 (84.20%)
Females	2 (4.3%)	6 (15.80%)
Body Mass Index	28.42 ± 4.56	27.53 ± 3.52
Time spent practicing regular physical activity, years	20.10 ± 11.27	18.09 ± 9.62
Time spent practicing rugby, years	16.48 ± 9.85	11.97 ± 7.10
IPAQ_Total; MET minutes/week	5484.18 ± 3626.83	8082.12 ± 6739.93
AAQ2_Total	23.85 ± 6.90	37.47 ± 7.76
ICFQ_Total	14.61 ± 4.62	25.37 ± 6.29
BC_Total	82.22 ± 11.08	74.87 ± 12.98
SF36_Global_Score	78.23 ± 7.90	65.27 ± 9.81

Note. ICFQ = Italian Cognitive Fusion Questionnaire; BC = Coping Orientation to Problems Experienced Inventory Italian version; AAQ2 = Acceptance and Action Questionnaire Italian version; SF36 = Short-Form Health Survey Italian version; IPAQ = Short Form of International Physical Activity Questionnaire Italian version.

## Data Availability

Collected data are available by contacting the corresponding author under proper request.
